# Exploring the IS-capades of Klebsiella pneumoniae: insertion sequences drive metabolic loss in obscure sub-lineages

**DOI:** 10.1099/mgen.0.001612

**Published:** 2026-01-22

**Authors:** Ben Vezina, Claire White, Helena B. Cooper, Kathryn E. Holt, Jane Hawkey, Kelly L. Wyres, Margaret M. C. Lam

**Affiliations:** 1 Department of Infectious Diseases, School of Translational Medicine, Monash University, Melbourne, Victoria, Australia; 2 Centre to Impact AMR, Monash University, Clayton, Victoria, Australia; 3 Department of Infection Biology, London School of Hygiene and Tropical Medicine, London, UK

**Keywords:** insertion sequence (IS), *Klebsiella*, metabolic modelling, metabolism, ozena, rhinoscleroma

## Abstract

**Introduction.** *Klebsiella pneumoniae* is an opportunistic pathogen that causes a wide spectrum of infections within healthcare settings and the community. Four *K. pneumoniae* sub-lineages, defined using core gene multi-locus sequence types, are known to cause distinct infections of the nasal and/or upper respiratory passages: SL91 and SL10031 (also referred to as subspecies *ozaenae*), SL10032 (subspecies *rhinoscleromatis*) and SL82. These sub-lineages have also demonstrated reduced carbon source utilization, which, in other species, has been linked with high loads of insertion sequences (ISs).

**Methods.** We performed comparative genomics, analysed IS composition and loads and constructed genome-scale metabolic models for available public sequences from these four sub-lineages. These were then compared with other sub-lineages from the wider *K. pneumoniae* population.

**Results.** The four focal sub-lineages displayed significantly higher IS loads (median range, 88–120 per genome) than other *K. pneumoniae* sub-lineages (median range, 12–73). Notably, each *K. pneumoniae* sub-lineage had unique IS profiles, consistent with distinct evolutionary trajectories of IS acquisition and expansion. Across sub-lineages, higher IS loads were inversely associated with the number of metabolic model genes per genome (R^2^=0.16; *P*<0.001), as well as predicted aerobic substrate utilization for phosphorus sources (R^2^=0.39; *P*<0.001), as per a second-degree polynomial regression model (*n*=1,664 genomes). Additionally, the four IS-dense sub-lineages displayed a combination of convergent, sub-lineage-specific substrate utilization losses, including the parallel loss of 3-phospho-d-glycerate, d-glycerate-2-phosphate and phosphoenolpyruvate utilization as carbon/phosphorus sources. Finally, inspection of IS insertion sites demonstrated frequent and non-destructive insertion next to transcriptional, carbohydrate and amino acid metabolism genes.

**Conclusions.** IS accumulation in *K. pneumoniae* was significantly associated with reduced metabolic substrate usage, consistent with an inverse relationship between IS load and metabolic capacity. Despite these losses, the affected lineages still demonstrate substantial metabolic breadth, consistent with early-stage, ongoing reductive evolution.

**Impact Statement**

*Klebsiella pneumoniae* is a highly diverse pathogen that is a leading cause of infections in healthcare and community settings. The overall population is comprised of many distinct, deep-branching lineages, each displaying variable traits (such as drug resistance and virulence) and that can be associated with unique disease pathologies. In particular, the *K. pneumoniae* lineages previously designated as subspecies *ozaenae* and subspecies *rhinoscleromatis* gained attention due to their nasopharyngeal-based pathologies. However, these obscure lineages are poorly understood despite their unique clinical characteristics. To explore these, we conducted comparative genomics and metabolic modelling on all isolates with available genomes dating back to 1920. Our study reveals that all lineages had unique insertion sequence (IS) profiles, and the nasopharyngeal lineages displayed significantly higher numbers of ISs than others. Metabolic modelling revealed that they had lost the ability to utilize nutrients otherwise conserved by the broader *K. pneumoniae* population; metabolic capacity was inversely associated with IS loads. These patterns are consistent with ongoing reductive evolution. This work provides the first detailed examination of these obscure but clinically distinct *K. pneumoniae* lineages.

## Data Summary

All data used in this study are available as Supplementary Material (Figs S1–S6, Tables S1–S3) at Figshare https://doi.org/10.6084/m9.figshare.30815960 [1]. Additionally, all analysis code is available at Figshare https://doi.org/10.6084/m9.figshare.28341917[Bibr R1]

## Introduction

*Klebsiella pneumoniae* is an opportunistic pathogen that causes a diverse range of infections within healthcare settings, such as pneumonia, bloodstream, surgical site and urinary tract infections. It is also a causative agent of infections in the community, associated with a unique subset of *K. pneumoniae* sub-lineages distinct from those that cause healthcare-associated infections [2]. As a species, *K. pneumoniae* exhibits a remarkable amount of genome diversity, with hundreds of unique ‘deep-branching’ sub-lineages and significant variation in its accessory genome content [2,3]. This diversity is likely an important driver of the different lifestyles and variable virulence profiles of *K. pneumoniae*.

Two sub-lineages of *K. pneumoniae*, clonal groups CG67 and CG90/91, based on seven-gene multi-locus sequence types (MLST) and formally designated as subspecies *rhinoscleromatis* and *ozaenae*, respectively, cause rare but unique chronic infections typically associated with the nasal and/or upper respiratory passages. Whole-genome sequencing has since clarified that both are divergent sub-lineages of *K. pneumoniae,* rather than separate subspecies as previously characterized [4]. The *K. pneumoniae* Life Identification Number (LINcode) taxonomic scheme (based on a 629-loci core genome MLST) assigns *K. pneumoniae* into >705 discrete sub-lineages [5]. Our sub-lineages of interest are SL10032 (previously *rhinoscleromatis* or CG67) and SL10031/SL91 (previously *ozaenae* or CG90/CG91). SL10032 strains often cause a chronic granulomatous disease described as rhinoscleroma, whereas SL91 and SL10031 have been reported to cause atrophic rhinitis or ozena. There have been at least 63 cases of *rhinoscleromatis* [6,10] and 45 *ozaenae* [9,23] infections documented in modern medical literature dating back to 1944, although there appears to be archaeological evidence of rhinoscleroma as far back as 300–600 AD from Maya (modern-day Guatemala) [24]. While these sub-lineages are usually associated with nasopharyngeal sites, there is historical evidence that they are able to colonize other body sites, including the urinary tract, soft tissue, blood, cerebrospinal fluid and gastrointestinal tract [12,13, 19, 22, 25, 26], although misidentification in the absence of whole-genome sequencing cannot be ruled out. Additionally, these sub-lineages have also been reportedly found in non-human hosts, isolated from cockroaches, cattle and chicken meat [14,22, 23].

Aside from their characteristic clinical presentations, these sub-lineages display a reduced metabolic capacity in biochemical tests compared with other *K. pneumoniae* sub-lineages [4,27], explaining why they were originally considered distinct sub-species. These sub-lineages, along with SL82 (ST82 or CG82 based on seven-gene MLST), have demonstrated reduced phenotypic carbon source utilization [4]. A link between this metabolic reduction and niche/pathogenic lifestyle adaptation has been posited [4]. This phenomenon has also been observed in other bacterial pathogens, such as *Shigella* species and *Bordetella pertussis* [28]. *Shigella*, in particular, is associated with the accumulation of insertion sequences (ISs) [29]. Reduced carbon utilization is also seen in SL82 [4], which, along with *ozaenae,* was unique among *K. pneumoniae* in lacking the *mrkD* type III fimbrial adhesin. SL82 also expresses the virulence-associated K1 capsule serotype. Similar to the other sub-lineages of interest, SL82 was noted to be strongly associated with respiratory infections (eight of 11 BioSample identifiers with sample metadata).

In this study, we performed a systematic analysis on a dereplicated collection of 1,664 completed and 210 draft *K. pneumoniae* genomes, to investigate the prevalence and impacts of IS on metabolic capacity in these four sub-lineages of interest. We hypothesized that, similar to *Shigella*, IS elements caused genome degradation and the loss of metabolic capabilities in these sub-lineages.

## Methods

### Genome acquisition, assembly and annotation

Complete *K. pneumoniae* genome assemblies (*n*=2,302) were initially downloaded from National Center for Biotechnology Information (NCBI) RefSeq (accessed on 04 July 2024; Table S1, available in the online Supplementary Material). To further expand genome numbers and improve the robustness of population-level inferences, 210 dereplicated NCBI BioSample IDs of target sub-lineages were obtained from Bacterial Isolate Genome Sequence Database (BIGSdb) [30] by selectively downloading genomes that matched the four sub-lineages of interest and their associated sequence types when no LINcode was determined, including SL10032 (ST67, ST3818, ST3819), SL91 (ST91, ST381, ST3766, ST3768), SL10031 (ST90) and SL82 (ST82, ST3764). Where available, short-read sequencing reads were downloaded from the Sequence Read Archive (SRA) (*n*=201), otherwise assembled sequences were downloaded from GenBank (*n*=20) (accessed on 29 October 2024; Table S1).

Short-read sequence data were assembled with Unicycler v0.5.1 [31]. Low-quality genomes were removed if they had >300 graphical fragment assembly dead ends or, in the absence of assembly graphs, an N50 of <65,000. This threshold was more lenient than previously defined quality-control metrics [32] to account for IS elements fragmenting short-read draft assemblies, resulting in larger contig numbers and dead-end counts. Remaining genomes (*n*=2,108) were then dereplicated using Assembly-Dereplicator v0.1.0 [33] with the following specifications: ‘--threshold 0.0003’. This resulted in a collective dataset of 1,874 dereplicated genomes. All genomes were annotated using Bakta v1.8.1 [34]. Pseudogene counts were extracted from the Bakta annotation .tsv file.

### Lineage assignment and genotyping

Dereplicated genomes were uploaded to Pathogenwatch (https://pathogen.watch/) for genotyping; namely, to confirm species and assign sub-lineage (SL), clonal groups and LINcodes [5,35]. A neighbour-joining tree was generated with PopPUNK v2.4.0 [36] as follows. The ‘create-db’ function was used with the following options: ‘--sketch-size 1000000 min-k 15 --max-k 29 --qc-filter prune’. The ‘fit-model’ function was subsequently used with the following options: ‘bgmm --ranks 1,2,3,5 --graph-weights --K 3’. Additionally, the ‘poppunk_visualise’ function was used, with the ‘--distances’ and ‘--previous-clustering’ parameters, to output a neighbour-joining tree. Kaptive v3.1.0 [37,38] was used to identify capsule synthesis loci or K-locus (KL), and genotyping was performed with Kleborate v3.1.3 [39].

### Metabolic model construction

Metabolic models were constructed using Bactabolize v1.0.3 [32] with the *Kp*SC pan v2.0.1 model [40] and the ‘--draft_model’ command. Growth across 1,278 conditions was simulated using the ‘--fba’ command on M9 minimal media, where positive growth was defined at a biomass threshold of ≥0.0001, as previously described [41].

### IS analysis

Plasmids were filtered out from the complete genome sequences using seqtk v1.3 [42], with the following command: ‘seq -L 4500000’, retaining only large sequences that are presumed to be chromosomal. Genomes were rotated to *dnaA* at base pair position 1 using rotate v1.0 [43], with the following command: ‘-s gtgtcactttcgctttggcagcagtgtcttgcccgattgcaggatgagtt -m 5’, to facilitate comparison of chromosomal synteny. Rotated, plasmid-free genomes (i.e. the chromosome) were used for the remaining analyses.

ISEScan v1.7.2.3 [44] was used to identify IS elements, which also identifies novel IS elements with divergence from known IS elements in the ISEScan database.

For comparisons within sub-lineage SL82, SKA v1.0.0 [45] was used to determine single nucleotide variants (SNVs) using ‘ska fasta’, followed by ‘ska distance -s 25’. Within sub-lineages, sub-lineage-specific phylogenetic minimum-evolution trees were constructed using SNV distances inferred from SKA, optimized via nearest-neighbour interchange with the ‘fastme.bal’ command from the R package ape v5.8 [46], then midpoint rooted via ‘midpoint.root’ from phytools v2.3–0 [47]. All-versus-all whole-genome alignments were performed using MUMmer4 v4.0.0 [48] using ‘nucmer -p’, and coordinates were extracted using ‘show-coords -d -l -T’.

Genome locus comparisons were performed by extracting gene coordinates via slice_multi_gbk.py (https://github.com/bananabenana/slice_multi_gbk), and Clinker v0.0.31 [49] was used for visualization.

### Statistical analysis and code

R v4.4 [50] and RStudio v2024.04.2+764 [51] were used for statistical analysis and visualization. R packages tidyverse v2.0.0 [52], ggtree v3.12.0 [53], colorspace v2.1–0 [54], ggpmisc v0.6.0 [55], ggpubr v0.6.0 [56], ggh4x v0.2.8 [57], rstatix v0.7.2 [58], gggenomes v1.0.0 [59] and patchwork v1.2.0 [60] were used. All R code can be found at Figshare (https://doi.org/10.6084/m9.figshare.28341917).

Linear regression was performed between IS loads and the ratio of nucleotides assigned to IS versus total chromosomal sequence. IS loads between sub-lineages were compared using a Kruskal–Wallis test with Holm adjustment for multiple comparisons, followed by a Dunn’s post-hoc test with Holm adjustment for multiple comparisons.

## Results

### Dataset description

To explore the impact of IS in *K. pneumoniae*, we utilized two dereplicated datasets: (i) a collection of complete genomes (*n*=1,664) from NCBI GenBank, used to quantify the exact numbers of IS within chromosomal sequences and sub-lineages; and (ii) an expanded dataset (*n*=1,874), which included targeted inclusion of draft assemblies. For the 1,664 complete genome dataset, only sub-lineages with ≥3 genomes after dataset dereplication were included, in an effort to maximize statistical power. For the expanded 1,874 genome dataset, additional genomes were acquired from key sub-lineages (see ‘Methods’). Both datasets included genomes from various sub-lineages, including, but not limited to, SL82, SL91-ozaenae, SL10031-ozaenae and SL10032-rhinoscleromatis. Throughout the manuscript, we will explicitly state which dataset was used. We found that 26 draft assemblies failed Pathogenwatch’s quality-control measures (Table S1), as they had >500 contigs, despite having high levels of genome completeness (≤300 graphical fragment assembly dead ends). We suspected that high contig numbers may be caused by large numbers of IS, and these assemblies were kept for analysis.

Significant diversity was observed in the expanded dataset (*n*=1,874), which was made up of 244 sub-lineages (SL) (based on the core gene multi-locus sequence type). To ensure appropriate population-level inferences, only sub-lineages with *n*≥3 genomes were included for further analysis, resulting in a dataset of *n*=1,441 genomes representing 65 sub-lineages. The majority of genomes were from sub-lineages associated with known multi-drug-resistant (MDR) (*n*=1,013 across 14 MDR SLs; 61.69%) or hypervirulent clones (*n*=129 from four hypervirulent SLs; 7.86%), as defined by Wyres *et al*. [2] (Table S1).

### Some sub-lineages display unusually high IS loads

**Fig. 1.**
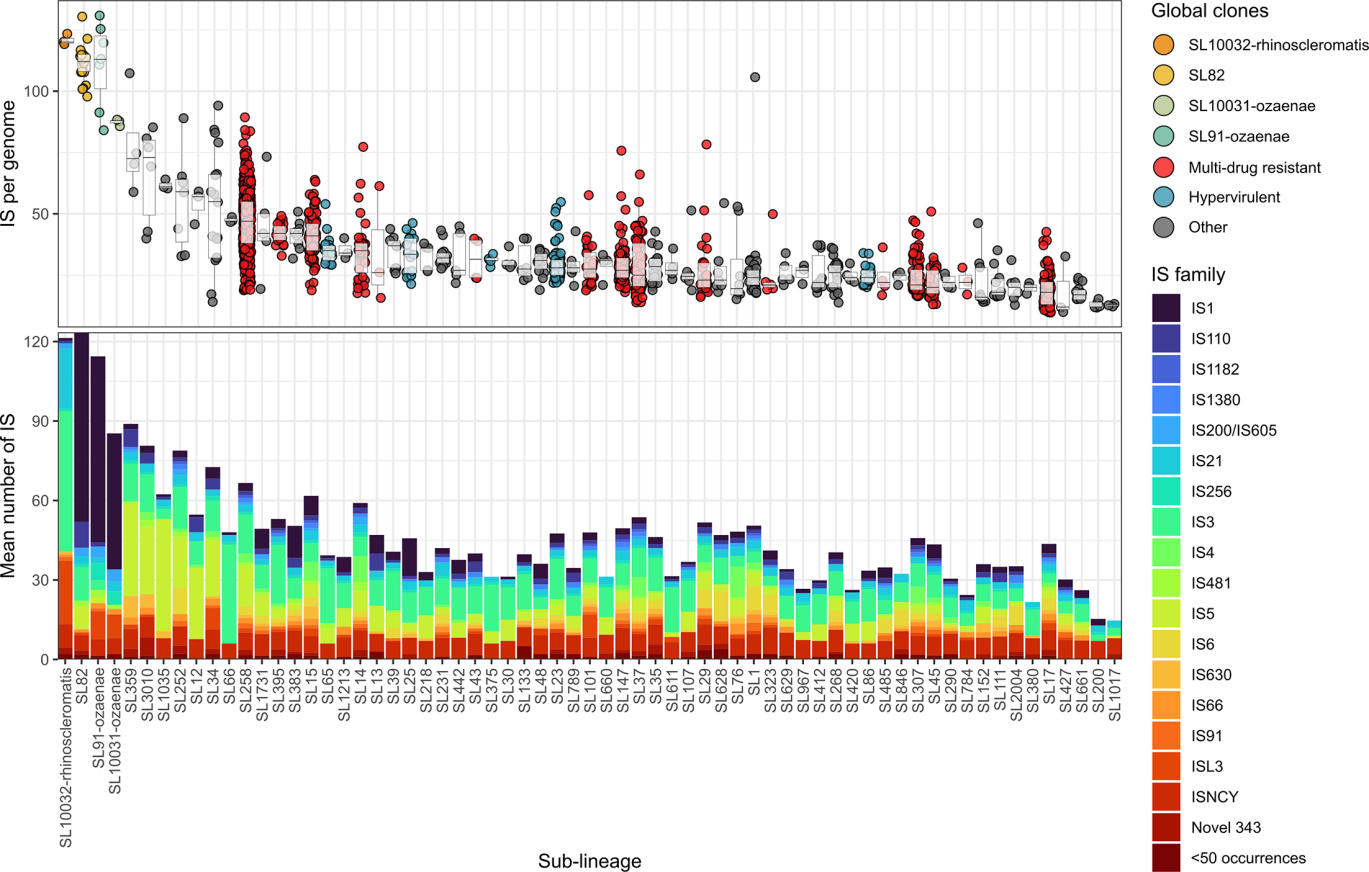
Sub-lineages display significantly different IS loads. Distribution of IS within 65 *K*. *pneumoniae* sub-lineages containing *n*≥3 complete genomes (*n*=1,441). The top panel shows the IS per genome, while the bottom panel shows the breakdown of IS families. Sub-lineages are coloured by their relevant global clone information [88], where each point represents a single genome. ‘<50 occurrences’ refers to IS families that were not shown in this figure due to their low abundance. Significance is not shown for brevity – raw data and results of Dunn’s post-hoc test are provided in Table S2.

Plasmid-free complete genomes (*n*=1,664) were screened for IS, and the numbers and types (i.e. IS families) were compared across the 65 sub-lineages with *n*≥3 genomes (Fig. 1). As expected, linear regression indicated a strong, positive correlation (R^2^=0.89; *P*<0.001) between IS loads and the ratio of nucleotides assigned to IS versus total chromosomal sequence (Fig. S1). Across each sub-lineage, the number of IS per genome (i.e. IS load) varied from 10 to 131 (median, 31). A total of 132 unique IS from 23 distinct IS families were detected across this dataset. Sub-lineages contained 3–20 distinct IS families, of which only three IS families (IS*3*, IS*21* and IS*NCY*) were found in every sub-lineage. Lineage-specific IS patterns were identified (Fig. S2, Table S2). Aside from five novel ISs (Novel 20, 24, 189, 272 and 348), which were detected in sub-lineages SL43, SL91, SL107, SL147 and SL17, respectively, no other IS families were restricted to a single sub-lineage. An additional 19 ISs were rarely detected across the population (i.e. found in <10 sub-lineages).

Sub-lineages SL82, SL91-ozaenae, SL10031-ozaenae and SL10032-rhinoscleromatis carried the highest IS loads, with median 88–120 ISs per genome, compared with other SLs (median, 12–73; Fig. 1). A Kruskal–Wallis test with Holm error correction indicated that sub-lineages had significantly different IS loads (*P*<0.0001). This was followed by a Dunn’s post-hoc test with Holm error correction, showing that SL82 had significantly more ISs per genome than 37 other sub-lineages (*P*<0.05), while SL91-ozaenae, SL10031-ozaenae and SL10032-rhinoscleromatis had significantly higher IS loads than 25, 5 and 6 other sub-lineages, respectively (Table S2). There were no significant differences between SL10032-rhinoscleromatis (median, 120±2 IQR), SL91-ozaenae (median, 113±21.5 Interquartile Range [IQR]), SL82 (median, 112±7 IQR) or SL10031-ozaenae (median, 88±1).

This statistical analysis also revealed that several other sub-lineages displayed significantly higher IS loads compared with others (Table S2). This included genomes that corresponded to MDR (SL258, SL15, SL395), hypervirulent (SL65) and common clones (SL34, SL3010), although there appeared to be within-clone variation in many sub-lineages. For example, the number of IS per genome within sub-lineage SL258 ranged from 22 to 80. Within-sub-lineage variability in IS loads was typically larger for sub-lineages represented by more genomes. With the exception of our four sub-lineages of interest, IS loads were generally similar across other clones, regardless of MDR or hypervirulence status.

### IS-dense sub-lineages are largely sourced from the human nasopharynx

Aside from isolates of unknown sampling site (*n*=42, 70% of genomes from the four sub-lineages of interest), the remaining isolates were recovered from human nasopharyngeal sites, including sputum (*n*=8), nasopharynx (*n*=6), maxillary sinuses (*n*=2), pleural cavity (*n*=1) and throat (*n*=1) (Table S1). One additional isolate was isolated from blood (*n*=1). While source metadata was missing for many of the SL10032-rhinoscleromatis and SL91-ozaenae isolates, sampling dates indicated collection between 1920 and 1952. Many samples collected during this period were generally of clinical origin. While there is likely a sequencing bias towards clinical isolates, these sub-lineages have previously been isolated from a variety of non-human sources, including chicken meat, cattle and cockroaches [14,22, 23], although no such isolates are currently represented in public genome repositories.

### IS profiles varied within IS-dense sub-lineages

**Fig. 2.**
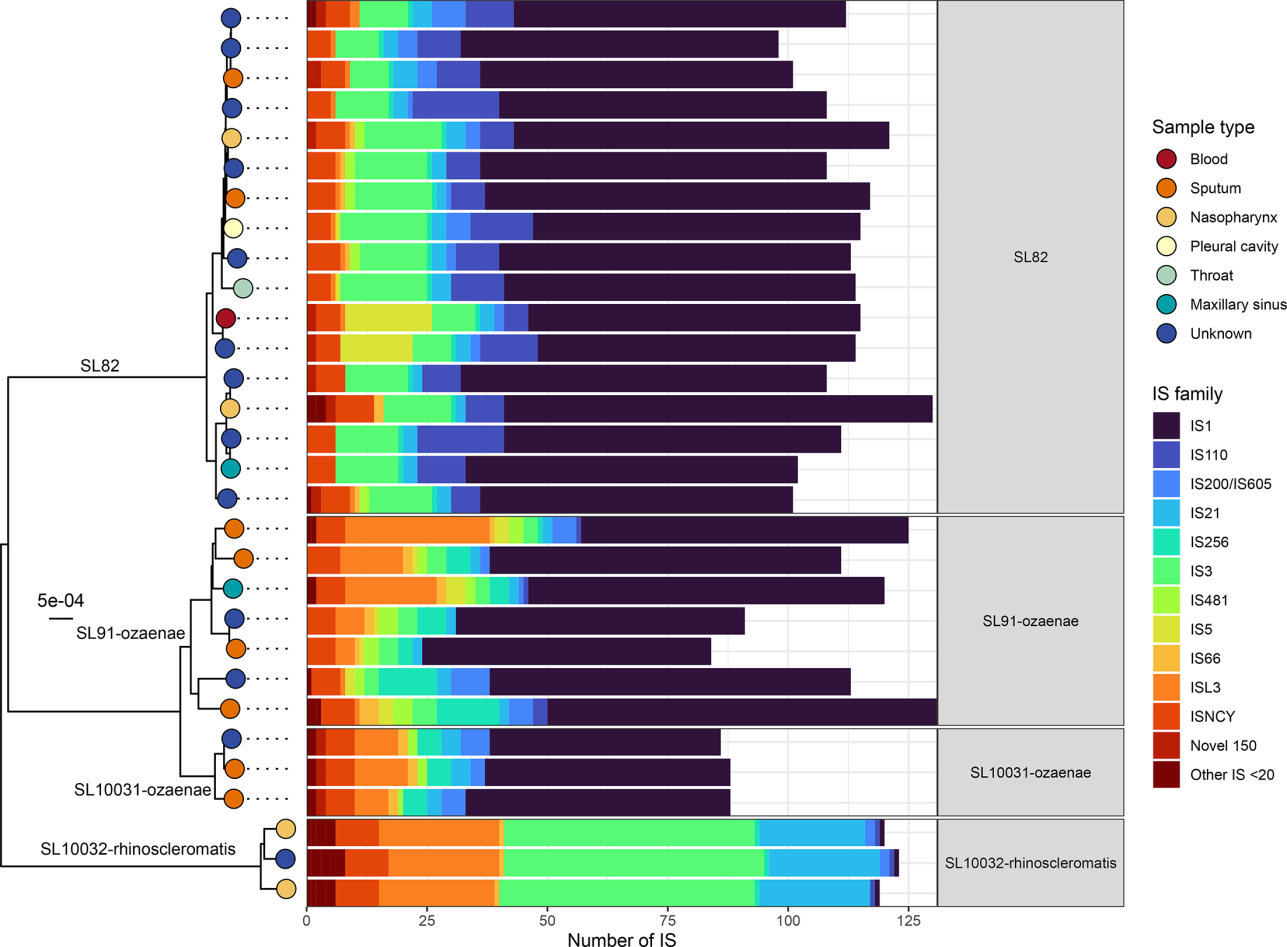
IS family prevalence differs between *K. pneumoniae* sub-lineages. Distribution of IS families across the four most IS-dense sub-lineages, aligned against a neighbour-joining tree generated from core k-mers, whereby each tip represents a unique genome and is coloured by the sample type. Only complete genomes with plasmids filtered out were used in this analysis.

The four focal IS-dense sub-lineages contained 16 (SL91-ozaenae), 15 (SL82), 13 (SL10031-ozaenae) and 10 (SL10032-rhinoscleromatis) IS families (Fig. 2). Of these, seven IS families (IS*1*, IS*L3*, IS*21*, IS*66*, IS*256*, IS*NCY* and IS*200*/IS*605*) were detected across all four lineages (Fig. S2). The IS profile of SL10032-rhinoscleromatis was the most distinct from the others, while the closely related *ozaenae* sub-lineages and SL82 shared highly similar IS profiles, consistent with their divergence from a more recent common ancestor in the core k-mer tree (Fig. S2). Most notable is the considerable expansion of IS*1* in SL82 (mean 71.53±6.31 sd copies per genome), SL91-ozaenae (70.14±7.9) and SL10031-ozaenae (51.3±3.5) genomes, while it was conversely rarer in SL10032-rhinoscleromatis (1±0). IS*1* expansion (mean ≥10 copies) was not specific to these sub-lineages and was found in six others, including hypervirulent SL25 and other clones SL10022 and SL383.

Notable differences were observed between the four focal sub-lineages, particularly regarding which IS families had undergone significant expansions. For example, expansion of IS*3* was observed in SL10032-rhinoscleromatis (mean 53±1 sd copies per genome) and SL82 (12.8±3.2), while SL91-ozaenae and SL10031-ozaenae carried fewer (3.7±1) or no IS*3*, respectively. IS*L3* was highly expanded in SL10032-rhinoscleromatis (24±1), SL91-ozaenae (10.57±10.81) and SL10031-ozaenae (9±2), but was largely absent in SL82 (1±0). In SL10032-rhinoscleromatis, IS*3*, IS*L3* and IS*21* comprised the largest proportions of IS (mean 53±1, 24±1 and 22.7±0.6 sd copies per genome, respectively), while carrying fewer IS*1* (mean 1±0 sd copies).

### IS load impacts strain metabolism and redundancy

We next analysed the impact of IS on metabolism across the entire dataset (complete and short-read-assembled genomes), although IS loads of short-read assemblies were not used in correlative measures. This included *n*=4 SL10032-rhinoscleromatis, *n*=15 SL91-ozaenae, *n*=5 SL10031-ozaenae and *n*=36 SL82. Metabolic models were built for all sub-lineages with *n*≥3 isolates, resulting in 1,874 genomes across 70 sub-lineages. Of these, 41 genomes from nine sub-lineages required minimal gap-filling to simulate growth on M9 media+d-glucose (mean 2.89±2.33 sd gap-filled reactions). SL34 was the most common lineage requiring gap-filling (*n*=4), likely caused by poorer draft genome assemblies. Gap filling is a standard process that is used to improve simulated growth prediction accuracy and minimize false-negatives [32].

Our model predictions were able to simulate 61/99 previously generated biochemical tests [4]. The models were unable to simulate the remaining 38 substrates, as these pathways are either not known or not encoded in the *Kp*SC pan-model v2. As such, we compared metabolic modelling growth predictions to these 61 phenotypic substrates to generate accuracy statistics. As many of the biochemically tested isolates do not have corresponding genome data, we compared model accuracy on a per-sub-lineage rather than individual basis to account for this [41]. F1 scores varied, ranging from 0.72 to 0.83 (Table 1). The inaccuracies were largely driven by false-positives (*n*=67 substrates), where the models predicted growth due to the presence of intact genes, while phenotypic tests predicted no growth, as seen by the high recall values ranging from 0.88 to 0.94. The small number of gap-filled genomes (*n*=41) did not influence the accuracy metrics observed.

**Table 1.** Summary of accuracy metrics of simulated metabolic model growth predictions. A total of 61 compatible phenotypic biochemical tests from [4] were used. Full data and confusion matrix can be found in Table S3

Sub-lineage	Accuracy	Precision	Recall	Specificity	F1 score	Biochemical test, *n*	Metabolic model, *n*
SL82	0.62	0.60	0.88	0.32	0.72	5	5
SL91-ozaenae	0.66	0.65	0.89	0.32	0.75	11	4
SL10031-ozaenae	0.66	0.63	0.94	0.30	0.75	9	36
SL10032-rhinoscleromatis	0.75	0.76	0.93	0.43	0.83	4	15

**Fig. 3.**
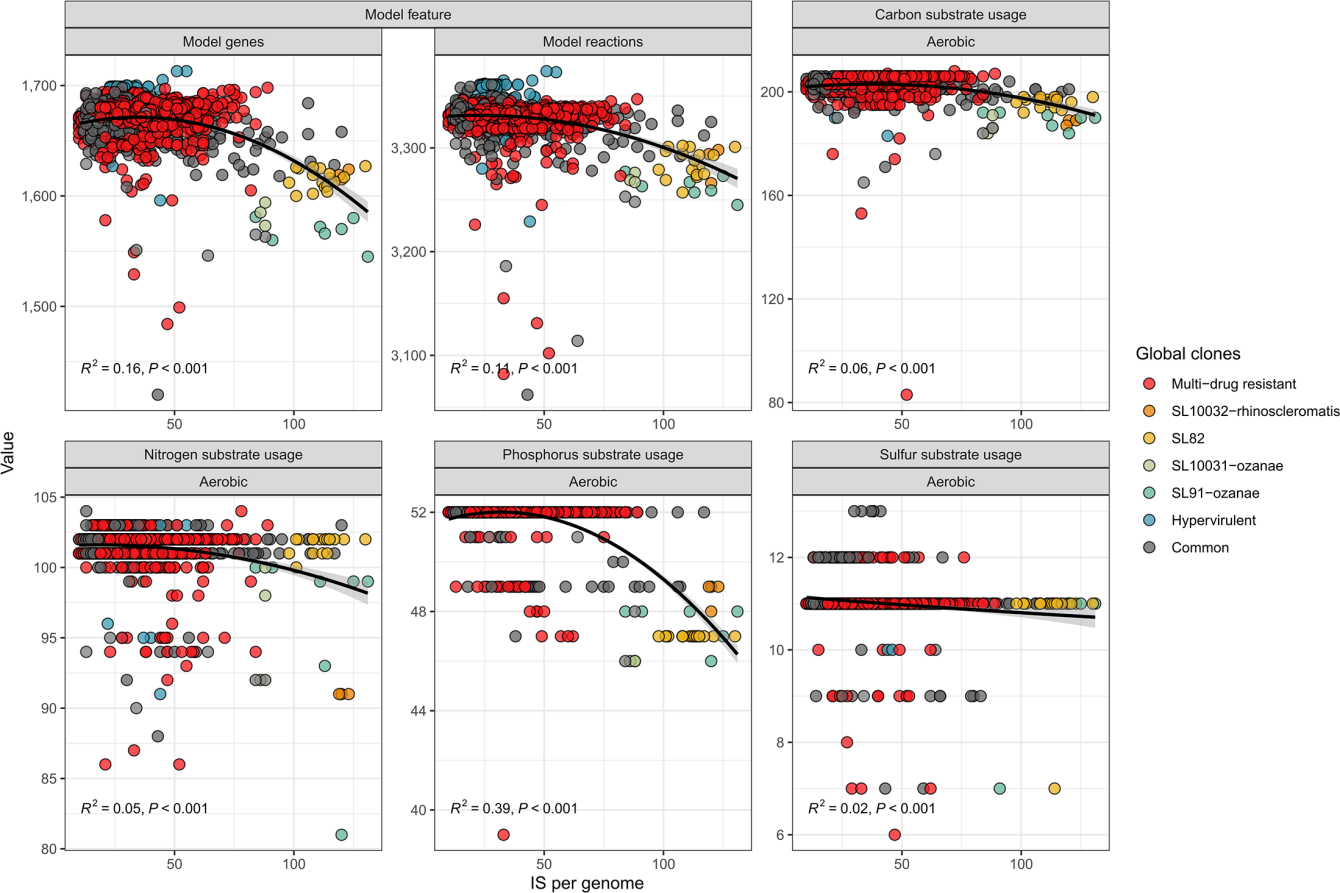
Number of IS per genome is related to loss of aerobic metabolic substrate usage. Number of ISs per genome along the *x*-axis, plotted against key metabolic model metrics derived from metabolic models along the *y*-axis. For model feature panels, the *y*-axis refers to the number of model genes or reactions per genome. For substrate usage panels, the *y*-axis refers to the number of substrates on which each model can grow. A squared polynomial model was fitted to the data. Each point represents a completed genome.

We next examined the association of IS load with strain metabolism. Analysis of only the completed genomes demonstrated a clear inverse relationship between IS load per genome and metabolic capacity, whereby high-IS sub-lineages displayed reduced metabolism (Fig. 3). Fitting a squared polynomial to the data, the number of model genes was inversely associated with IS load (R^2^=0.16; *P*<0.001) and slightly less so with model reactions (R^2^=0.16; *P*<0.001). For substrate usage, only aerobic use of phosphorus sources displayed a moderate inverse relationship (R^2^=0.39; *P*<0.001). There was a null-strength association between IS load and sulphur substrate usage, likely also impacted by the low number of absolute substrates that could be simulated (maximum of 13 different sulphur sources). The association between IS loads and anaerobic usage of carbon, nitrogen, phosphorus and sulphur substrates was weaker when compared with aerobic substrate usage of the same compounds. This was likely driven by either the reduced predictive capacity of anaerobic growth [40] and/or the reduced substrate usage capacity of *K. pneumoniae* under anaerobic conditions [41].

The metabolism of some over-represented sub-lineages, such as the MDR global clone SL258 (*n*=427), was unaffected by IS load (R^2^ <0.01; *P*<0.263 to *P*<0.96) despite a wide IS range (19–89) (Fig. S3). To control for this over-representation effect, we calculated mean values for each sub-lineage and re-examined the relationships (Fig. S4). Increasing IS load was now weakly associated with reduced aerobic use of carbon (R^2^=0.23; *P*<0.001) and nitrogen (R^2^=0.13; *P*<0.001), and moderately associated with phosphorus (R^2^=0.42; *P*<0.001) sources, using a squared polynomial model. The reduced metabolic capacity of sub-lineages with higher IS loads was consistent with the similar inverse relationships of the total number of metabolic model genes (R^2^=0.31; *P*<0.001) and model reactions (R^2^=0.25; *P*<0.001). There was no correlation between IS load and the number of pseudogenes (R^2^ <0.01; *P*=0.775), which was also previously reported in *Shigella* species [29].

**Fig. 4.**
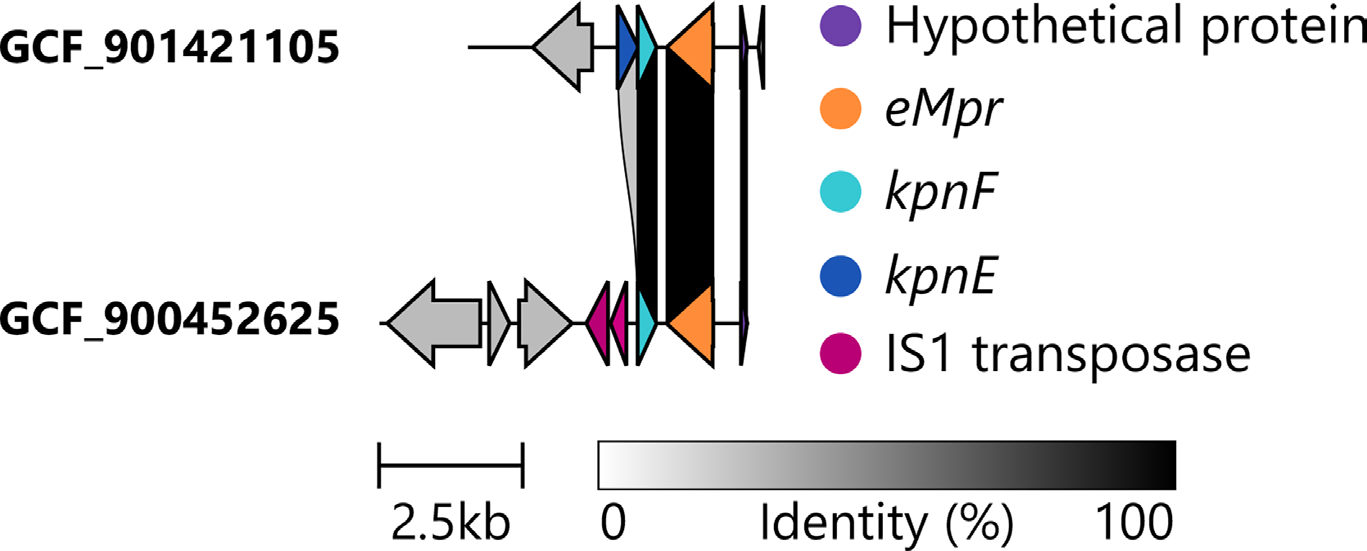
IS*1* results in complete loss of the *kpnE* gene, required for the *KpnEF* spermidine antiporter, in an SL82 genome (accession GCF_900452625). Identity percentage between genes is shown by greyscale links.

The pan KpSC metabolic model [40], which was used as a reference model in this study, contains genes (i.e. reference model genes) that encode metabolic reactions. To determine whether any of these were interrupted by IS and present as genetic remnants, the complete genomes from SL82, SL91-ozaenae, SL10031-ozaenae and SL10032-rhinoscleromatis were screened. A total of 232 reference model genes were absent from these genomes and comprised a combination of core and variable model genes, as previously defined in a large-scale metabolism study [41]. Briefly, if a model gene was found in ≥95% of all genomes, it was considered a ‘core’ model gene, while model genes that were found in <95% of isolates were considered ‘variable’. Flanking sequences of up to 50 bp around IS sites were matched against missing model genes to determine if IS had interrupted them directly. Only five genes, across 16 total occurrences, showed evidence of direct IS interruption, where the same gene was often found interrupted in multiple closely related genomes. This meant that 1.5% of missing model genes can be directly attributed to a known IS insertion. For example, *kpnE*, an essential component of the spermidine antiporter complex KpnEF, was interrupted by IS*1* within a single SL82 genome (accession GCF_900452625, coordinates 2591281 : 2597710 on contig 1), resulting in complete loss of the *kpnE* sequence (Fig. 4).

Notably, the higher IS loads were significantly uncorrelated with genome size (Fig. S1) nor with pseudogene counts (Figs S5 and S6).

### Metabolic evolutionary parallelism

**Fig. 5.**
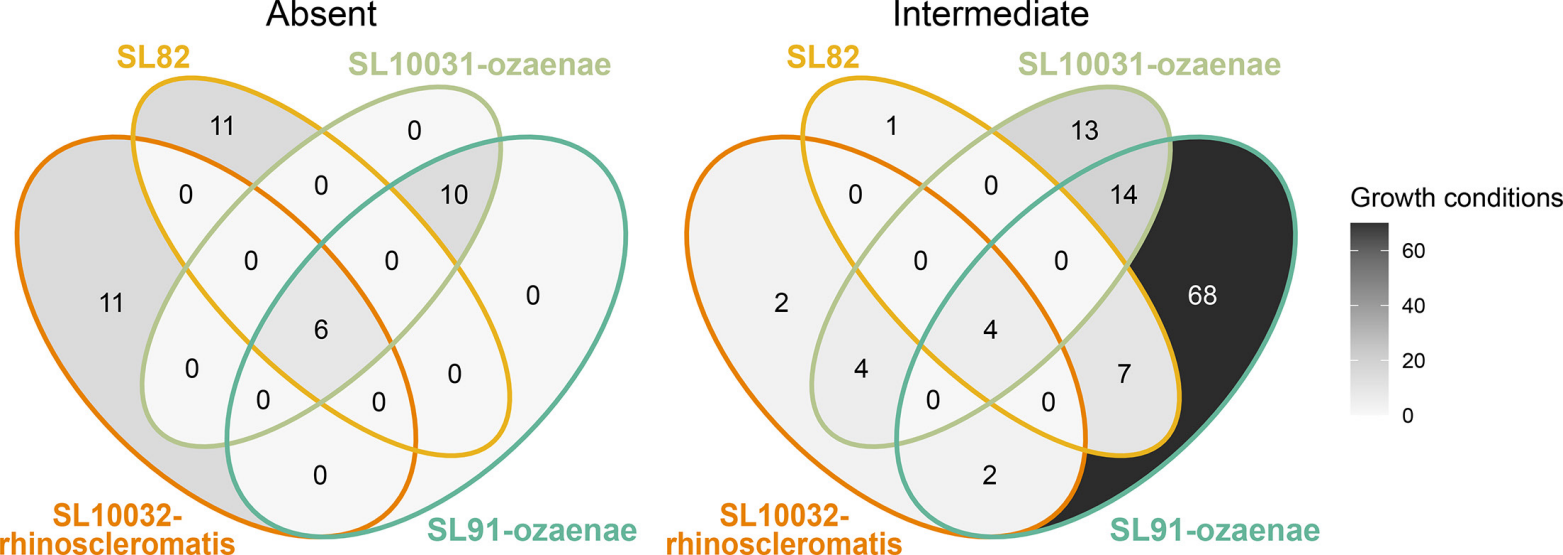
Sub-lineages display unique and convergent metabolic losses. Venn diagrams showing the number of *K. pneumoniae* core substrate usages that are either intermediately (usage within >0% and <95%) or completely lost (usage within 0%) within sub-lineages.

Aside from their loss of total substrate usage, these sub-lineages displayed distinct but convergent metabolic trajectories. Previously, 616 core metabolic traits were found to be conserved across the 48 most prevalent *K. pneumoniae* sub-lineages from a dataset comprising genomes from multiple large-scale studies (4,621 genomes; *n*≥15 genomes in each) [41]. By raw counts, each of the genomes from the four focal sub-lineages in our analysis appears to have completely lost the ability to utilize 15–20 core *K. pneumoniae* substrate growth conditions. Genomes within the four focal sub-lineages also demonstrated variable, intermediate loss of 12–49 substrates, with only some genomes were predicted to have lost the ability to grow on particular substrates (Fig. 5, Table S3).

There was evidence of convergent substrate utilization losses, whereby all four sub-lineages have lost the ability to use 3-phospho-d-glycerate, d-glycerate-2-phosphate and phosphoenolpyruvate as carbon or phosphorus sources. This was due to the loss of a periplasm antiporter *pgtP* and the associated operon *pgtABC*, responsible for the import of these substrates from the periplasm to the cell. Due to the complete contextual loss of this operon and numerous intra-genomic rearrangements, it is not clear whether this loss was ISs-mediated. As expected, based on their genetic relatedness, SL91-ozaenae and SL10031-ozaenae displayed ten conserved substrate losses, likely as a result of common ancestry. Despite similar IS profiles between SL82 and the two ozaenae sub-lineages, they displayed little overlap of metabolic losses. Sub-lineage-specific complete metabolic losses included SL82’s loss of 3-hydroxyphenylacetic acid usage as a carbon source; SL10032-rhinoscleromatis’s loss of 5-aminopentanoate, l-lysine and 4-aminobutanoate usage as carbon and nitrogen sources; and SL91-ozaenae/SL10031-ozaenae’s loss of (R)-3-hydroxybutanoate, cytosine and myo-inositol hexakisphosphate usage as carbon and nitrogen sources (Table S3).

### Virulence and capsule characteristics

Siderophore-encoding virulence genes associated with iron uptake were widely detected within these sub-lineages, with 29/60 genomes displaying Kleborate virulence scores ≥3 (i.e. indicating the presence of aerobactin, with or without yersiniabactin, encoded by *iuc* and *ybt*, respectively) [39] (Table S1). The *ybt* locus, which is typically chromosomally encoded on an integrative and conjugative element (ICE) called ICE*Kp*, was found in 3/4 SL10032-rhinoscleromatis genomes (lineage *ybt* 11 on ICE*_rhino*), 13/15 SL91-ozaenae (lineage *ybt* 10, ICE*Kp*4) and 2/5 SL10031-ozaenae (lineage *ybt* 16, ICE*Kp*12), although the locus was truncated in 12/18 cases across this dataset due to contig breaks. The *iuc* locus was found in all SL10032-rhinoscleromatis (lineage *iuc* 4, chromosomal, 1/4 truncated), 6/15 SL91-ozaenae (*iuc* 2A, plasmid), 2/5 SL10031-ozaenae (*iuc* 2A, plasmid) and 17/36 SL82 (*iuc* 2A, plasmid). Salmochelin (encoded by *iro,* and its presence or absence not considered in the Kleborate virulence score) was found in 17/36 SL82 as novel *iro* alleles. Previously reported *mrkD* (type III fimbriae adhesin) [4] was completely absent in all focal sub-lineages, with the exception of SL10032-rhinoscleromatis (present in 5/5 genomes). The *rmpADC* locus, which controls capsule expression and hypermucoviscosity, was also commonly detected in genomes from these four sub-lineages. It was found in 32/36 SL82 genomes (lineage *rmp* 2A, truncated in 26 genomes), all SL10032-rhinoscleromatis genomes (*rmp* 4, truncated in one genome) and 1/15 SL91-ozaenae (*rmp* 2A, truncated). These virulence gene truncations (*ybt*, *iuc*, *rmp*) were associated with the ends of assembly contigs, likely caused by IS insertions.

All SL82, SL10031-ozaenae and SL10032-rhinoscleromatis genomes had typeable and homogenous capsular loci (KL1, KL5 and KL3, respectively), consistent with previous reports [4] based on capsule typing. In contrast, SL91-ozaenae isolates displayed varying K loci, with closest matches to KL4 (*n*=13), KL6 (*n*=1) and KL1 (*n*=1), with all but the KL6 genome being untypeable – again due to capsule gene truncations and fragmented assemblies, likely caused by IS insertions within the capsule loci, which has been previously reported in *K. pneumoniae* [61]. As public assemblies lacked reads, it was not possible to leverage assembly graphs to determine which IS caused contig breaks – although in complete genomes such as GCF_014218685.1, IS*1* was responsible for the capsule null predictions. However, 12 genomes were predicted to be capsule null as a result of capsule locus interruptions, including 2/5 SL10031-ozaenae, 3/15 SL91-ozaenae and 7/36 SL82.

### IS variation within sub-lineages confers strain-level metabolic diversity

**Fig. 6.**
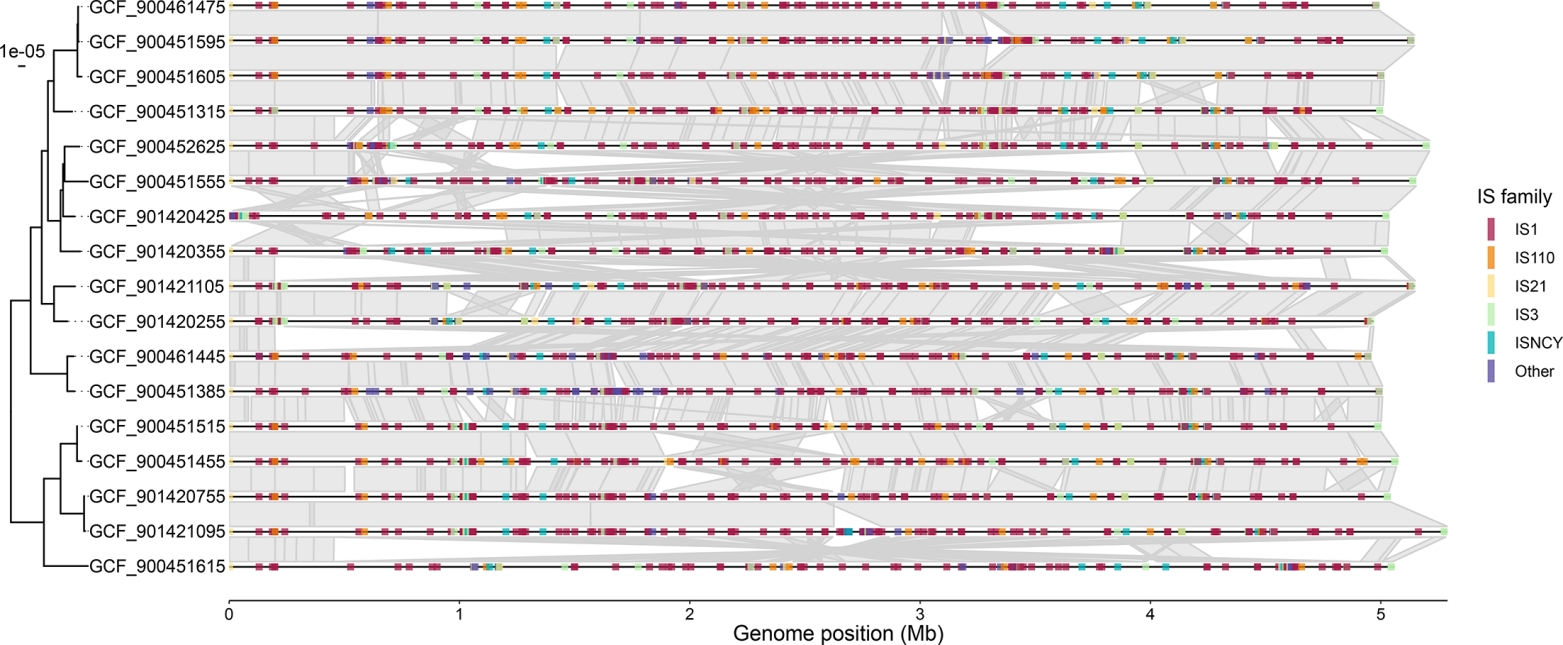
ISs vary locationally within SL82. Comparison of IS and homologous blocks between SL82 complete chromosomes. The tree shown is a SNV distance tree showing 17 genomes. Grey links between genomes represent homologous sequence blocks as aligned by nucmer with standard parameters. Coloured lines show IS families. All genomes start at *dnaA*.

To study the localized impact of IS further, we analysed all available SL82 genomes (*n*=17), as this sub-lineage had the largest number of complete genomes. After re-orientating the genomes to the same starting position, we mapped IS across the sequences and inspected IS insertion sites and chromosomal synteny (Fig. 6). While many of these SL82 genomes shared similar IS profiles and IS density, no two genomes were identical with respect to IS insertions, highlighting the dynamic nature of IS and the consequent losses or gains of DNA segments. Additionally, chromosomal inversions and rearrangements were common, which were often flanked by ISs, reasonably explaining the rearrangements occurring at homologous repeat sites.

Genomes within SL82 were predicted to have small but variable aerobic metabolism across 24 substrates, with 13/17 genomes showing loss of usage for at least one substrate. The most variable substrates were 4-hydroxybenzoate (usage observed in 7/17 genomes), l-alanine-d-glutamate-meso-2,6-diaminoheptanedioate (13/17 genomes) and l-galactonate (15/17 genomes). The remaining 21 substrates were absent only in a single genome (16/17). While we do not have matching phenotypic and genomic data, predictions for 4-hydroxybenzoate matched previously generated phenotypic data (Table S3).

## Discussion

Reduced metabolic versatility has previously been documented in select sub-lineages of *K. pneumoniae*: SL10032-rhinoscleromatis, SL10031-ozaenae, SL91-ozaenae and SL82 [4]. This is thought to be an important driver of their unique ecological and pathogenic profiles, particularly as these strains are often isolated from the upper respiratory tract but are by no means restricted to these sites or human hosts [14,22, 23]. In this study, the application of genome-scale metabolic modelling supported a significant loss of substrate usage across SL82, SL10031-ozaenae, SL91-ozaenae and SL10032-rhinoscleromatis compared with other *K. pneumoniae* sub-lineages. In particular, these sub-lineages appear to have lost the ability to utilize gut microbe-/human-derived metabolites for energy, including 3-phospho-d-glycerate, d-glycerate-2-phosphate, phosphoenolpyruvate, 3-hydroxyphenylacetic acid and 5-aminopentanoate [62,66], along with myo-inositol hexakisphosphate found in plant tissues and human diet [67] and human metabolites (R)-3-hydroxybutanoate (liver) [68] and 4-aminobutanoate (neurotransmitter) [69]. These findings hint that these sub-lineages have deviated from other *K. pneumoniae* sub-lineages. The genome sizes of these four focal sub-lineages have not decreased compared with other *K. pneumoniae* Species Complex (*Kp*SC) lineages (Figs S5 and S6), and their metabolic capacities remain quite substantive, retaining the use of 552–587 out of 615 ‘core *Kp*SC metabolic traits’ [41]. These observations do not quite align with what one would expect in the advanced stages of reductive evolution and niche specialization [70,72]. Coupled with the detection of these four sub-lineages in various non-nasopharyngeal [12,13, 19, 22, 25, 26] and non-human sources [14,22, 23], this pattern suggests early-stage, ongoing reductive evolution that may be driving a gradual specialization towards a nasopharyngeal niche.

While all *Kp*SC appear to both readily colonize the gut prior to infection [73] and have an intracellular lifestyle phase [74,77], which has been specifically demonstrated in SL10032-rhinoscleromatis in an animal model [78], the loss of key metabolic features in SL10031-ozaenae, SL91-ozaenae, SL10032-rhinoscleromatis and SL82 may be consistent with adaptation to an intracellular lifestyle. It is well-established that the capsule is dispensable within the *Kp*SC intracellular phase and enhances intracellular survival in macrophages and neutrophils [74,75], consistent with our findings of capsule loss in SL10031-ozaenae, SL91-ozaenae and SL82. In addition, inflammatory monocytes are recruited in response to SL10032 infections [78], which are known to be activated by glycolysis [79]. Core glycolysis intermediate products, including 3-phospho-d-glycerate, d-glycerate-2-phosphate, phosphoenolpyruvate and the pentose phosphate pathway-inducer d-glucose 1-phosphate, are lost in these sub-lineages. Additionally, single-cell RNA-seq experiments have demonstrated that macrophage polarization is driven by glycolysis as a result of *K. pneumoniae* infections [80]. This, in conjunction with the moderate rates of predicted capsule null genomes, provides a prospective model of host-adapted intracellular specialization in these obscure lineages.

The presence of known virulence determinants (*ybt, iuc, rmp* and *iro*) varied across the focal sub-lineages. It is unclear whether the presence of *iuc* and *rmp* confers hypervirulence, given that these lineages and their associated mobile elements are distinct from the typical large *K. pneumoniae* virulence plasmid (KpVP-1 with *iuc1, rmp1, iro1*) observed in hypervirulent clones such as SL23. Notably, some hypervirulent lineages, such as SL66 (KpVP-2 plasmid with *iuc2, rmp2, iro2*), also displayed slight median increases in IS loads. Associations between virulence factors and IS have previously been found in other species [81].

We demonstrate for the first time that these four sub-lineages also have significantly higher IS loads (Fig. 1), which has been linked to streamlining of metabolic profiles in *B. pertussis* and various *Shigella* species [28,29]. IS has previously been quantified within *K. pneumoniae* SL258 (ST258) [82], which found a stable repertoire of IS elements and small within-lineage variation (specifically, an increase in IS load in one subclade of SL258, referred to as ST258B). This is consistent with the IS profiles and within-sub-lineage variation that were observed in our study (Figs1  2, Table S2), such as the intermediate, rather than complete, loss of core *K. pneumoniae* substrates within a sub-lineage (Fig. 5).

Notably, the limited impact of IS on metabolism in one IS-rich subclade of SL258 (previously designated as subclade ST258B [82]) contrasted with the patterns observed in the four focal sub-lineages. This variance indicates that *K. pneumoniae* sub-lineages have different relationships and IS-mediated evolutionary trajectories. This may also reflect some degree of insertional tolerance, whereby IS insertions are not detrimental to survival. SL91-ozaenae and SL10031-ozaenae are closely related to each other and to SL82 (Fig. S2), as evidenced by their shared IS profiles and the major expansion of IS*1* observed across all three sub-lineages (Fig. 2). We speculate that their common ancestor had already initiated expansion of IS, followed by metabolic gene losses. These inferences, however, are limited by sampling biases in public data and the relative rarity of these sub-lineages.

Direct IS interruptions of metabolic genes were detected in only a few instances (only five of 323 examples were found). We hypothesize that ISs were responsible for the initial disruption of some key metabolic genes, with these disruptions being selectively tolerated at the time of transposition, followed by degradation or loss of the affected loci over time. The inverse relationship between IS load and substrate usage, particularly aerobic carbon and phosphorus utilization (Figs 3 and S4), supports this hypothesis. In many cases, the initial IS insertion and gene interruption may have been subsequently purged via recombination, rearrangement or genome degradation. Additionally, higher IS loads potentiate a higher rate of genomic rearrangement [83], deleting genes without direct insertion. These observations highlight that DNA sequence analysis of isolated pathogens cannot capture the *in vivo* conditions or genetic history that have shaped the organism, even when historical isolates are used. Hawkey *et al*. [29] noted significant genome degradation from IS insertions in *Shigella* species, which caused metabolic usage losses. We did not observe any correlation between IS load and genome length in our dataset, contrasting with *Shigella,* where the two appeared to be inversely related. The metabolic gene losses in both *Shigella* and the four *K. pneumoniae* sub-lineages of interest provide insight into the cellular processes required for these pathogens to colonize and progress to human disease. Our data show that, while these sub-lineages displayed loss of some *K. pneumoniae*-specific core traits, they retained use of 552–587 core metabolic traits. Given the conservation of these core metabolic traits across all *K. pneumoniae* sub-lineages, these are likely key to the species’ ability to colonize and/or cause infections.

Metabolic inferences are caveated by a lack of direct phenotypic validation of the isolates from which genomes were obtained, leading to population-level accuracy metrics (Table 1). As it currently stands, the metabolic models have broad agreement with previously published biochemical tests [4,27], whereby these IS-dense sub-lineages exhibited considerable loss of metabolic potential (Fig. 5), but further metabolic phenotyping is required to confirm this. The decaying polynomial relationship between the increased number of ISs per genome and loss of substrate growth conditions, as well as metabolic genes and reactions (Fig. 3), demonstrates this effect. Lower accuracy scores were observed (Table 1) and can be explained by various reasons. False-positives are not always indicative of model inaccuracies, but usually indicate either the presence of metabolic genes that failed to be expressed under the experimental conditions due to gene regulation [41,84], or the absence of intact copies of key genes in the phenotypically tested isolates. Discrepancies likely stem from the strains used in biochemical tests, which may not be reflective of the larger population. In some cases, false-positives could be explained by ISs interrupting the promoter/regulatory regions required for expression to occur. For example, the genes required for l-histidine, l-tyrosine, ethanolamine, quinate, 2-ketoglutarate and putrescine utilization are all present in the genomes but phenotypically showed no growth. Alternatively, if these false-positives did not arise from gene regulation issues, they may reflect incorrect assumptions in the metabolic models, such as over-assignment of metabolic genes. A metabolic model gene is considered ‘present’ if there is ≥25% bi-directional coverage (standard value) [32,40, 85]. It is therefore possible that our model-based analysis is underestimating the impact of IS in reducing the metabolic capacity of isolates. It is also possible that the four focal IS-dense sub-lineages may contain additional novel or specialized metabolic reactions that are not accounted for in the current model.

Insertion of ISs not only leads to genomic rearrangements, as observed in SL82 (Fig. 6), but has also been shown to impact the expression of neighbouring genes via promoter readthrough, such as IS*1* [86,87]. Considering the vast numbers of ISs found across these IS-dense sub-lineages and their close proximity to many regulatory, amino acid and carbohydrate metabolism loci (Fig. S2), it may be that ISs are facilitating metabolic specialization via provision of a selective advantage to organisms by transcriptionally upregulating remaining metabolic traits, in addition to purifying unneeded metabolic traits. For example, a copy of IS*1* was found directly upstream of the *cra* catabolite repressor/activator gene in an SL82 genome (accession GCF_900451215), which regulates the acetolactate *ilvIN* locus responsible for amino acid biosynthesis from pyruvate. Increasing expression of *cra* may provide a selective advantage for SL82 and balance the effects arising from usage loss of 17 core substrates.

Overall, our findings demonstrate that high IS loads are a key distinguishing feature of the four nasopharyngeal-associated *K. pneumoniae* sub-lineages and are broadly linked to reduced metabolic substrate utilization. Despite these losses, the retention of substantial metabolic capacity suggests that these lineages are somewhere along the reductive evolution trajectory rather than exhibiting advanced genome degradation. Future work integrating transcriptomics and experimental validation will be important for resolving the functional consequences of IS insertions and their role in the evolutionary specialization of these sub-lineages.

## Abbreviations

ISs: insertion sequences
SNVs: single nucleotide variants
